# Indocyanine green fluorescence-guided laparoscopic hepatectomy versus conventional laparoscopic hepatectomy for hepatocellular carcinoma: A single-center propensity score matching study

**DOI:** 10.3389/fonc.2022.930065

**Published:** 2022-07-19

**Authors:** Wang Jianxi, Zou Xiongfeng, Zheng Zehao, Zhao Zhen, Peng Tianyi, Lin Ye, Jin Haosheng, Jian Zhixiang, Wang Huiling

**Affiliations:** ^1^ The Second School of Clinical Medicine, Southern Medical University, Guangzhou, China; ^2^ Department of General Surgery, Guangdong Provincial People’s Hospital, Guangdong Academy of Medical Sciences, Guangzhou, China

**Keywords:** indocyanine green, laparoscopy, hepatectomy, outcomes, hepatocellular carcinoma

## Abstract

**Background:**

Indocyanine green fluorescence-guided laparoscopic hepatectomy (ICG-guided LH) is increasingly used for the treatment of hepatocellular carcinoma (HCC). However, whether ICG-guided LH can improve surgical outcomes remains unclear. This study aimed to investigate the short-term outcomes and survival outcomes of ICG-guided LH versus common laparoscopic hepatectomy (CLH) for HCC.

**Methods:**

We conducted a retrospective analysis of 104 ICG-guided LH and 158 CLH patients from 2014 to 2020 at our center. To avoid selection bias, 81 ICG-guided LH and 81 CLH cases were analyzed after 1:1 propensity score matching (PSM). The baseline data and results were compared between the two groups.

**Results:**

The baseline characteristics of both groups were comparable after matching. There was a significant difference in operative time: longer in the ICG-guided LH group than in the CLH group (p=0.004). However, there was no significant difference in operative time in anatomical resection between the two groups (p=0.987). There was a significant difference in operative time in non-anatomical resection: longer in the ICG-guided LH group than in the CLH group (p=0.001). There were no significant differences in positive surgery margin, blood loss, blood transfusion rate, postoperative complication rate, postoperative length of hospital stay, mortality within 30 days, and mortality within 90 days. The ICG-guided LH group appeared to have a trend towards better overall survival (OS), but there was no significant difference in OS (P=0.168) and recurrence-free survival (RFS) (P=0.322) between the two groups.

**Conclusions:**

Although ICG fluorescence-guided LH is a timelier procedure to perform, it is a safe and effective technique with the advantages of intraoperative positioning, low postoperative complication rates, and potential to improve OS.

## Introduction

Hepatocellular carcinoma (HCC) was one of the most common cancers worldwide in 2020 (1). Although new drugs and trials for advanced HCC have developed rapidly in the past few years (2), there are still limited methods to cure early HCC, which include liver resection, liver transplantation and local ablation therapies. Unfortunately, local ablation therapies have been reported to have shorter recurrence-free survival (RFS) than liver resection (3), and liver transplantation is limited, leaving liver resection as the primary radical treatment for early HCC. In the past ten years, the application of laparoscopic hepatectomy (LH) has been popular all over the world (4), especially in large expert centers. In contrast to open hepatectomy, LH seems to have similar oncological survival outcomes, shorter hospital stay, less blood loss and less operative morbidity (5, 6). To better judge the boundary of the tumor and the extent of resection during laparoscopic surgery, indocyanine green (ICG) fluorescence navigation has been applied during laparoscopy. Through intraoperative real-time fluorescence navigation, R0 resection can be achieved, and the tumor can be completely removed while preserving as much of the liver as possible. ICG fluorescence navigation also has a positive effect on the detection of small liver cancer (7). ICG fluorescence navigation can help identify the ductal system of the liver during surgery, which may help identify intraoperative bile leakage and reduce the occurrence of postoperative bile leakage (8, 9).

However, whether indocyanine green fluorescence-guided laparoscopic hepatectomy (ICG-guided LH) or liver resection have an advantage in survival rate remains unclear. Previous studies have suggested less blood loss, less intraoperative blood transfusion rate, shorter operation time, shorter hospital stay and lower complication rate during surgery with ICG fluorescence navigation (5, 6, 10–14). These studies mostly involved only a few cases and lacked propensity score matching (PSM) and prognostic data (15). Several meta-analyses have been conducted on this topic. Qi et al. (16, 17) reported that the ICG fluorescence imaging-guided hepatectomy group had different results in operative time, blood loss, blood transfusion rate, hospital stay and postoperative complications compared with the traditional hepatectomy group. Yu Liu et al. (18) showed that ICG fluorescence imaging-guided hepatectomy shortened operative time, reduced intraoperative bleeding, shortened hospital stay and lowered postoperative complication rates. In summary, ICG-guided LH was superior to non-ICG imaging-guided hepatectomy. Furthermore, additional matched cohorts and systematic reviews should be conducted to support these findings.

To provide more evidence in ICG-guided LH, we conducted this study using 1:1 PSM to compare the short-term outcomes and survival results of ICG-guided LH versus common laparoscopic hepatectomy (CLH) for HCC.

## Methods

### Study participants

We retrospectively collected data from patients pathologically diagnosed with HCC. A total of 289 patients, including 173 CLH and 116 ICG-guided LH cases, were enrolled in this study. All patients underwent LH from 2014 to 2020 in the Guangdong Provincial People’s Hospital with an ECOG-PS score of 0-2. Patients who underwent ICG-guided laparoscopic non-anatomical hepatectomy received an intravenous injection of 0.5 mg/KG ICG 3-5 days before surgery (**Video 1**). In contrast, patients who underwent ICG-guided laparoscopic anatomical hepatectomy were either injected with 5-10 ml of ICG (0.025 mg/ml) into the tumor-feeding portal branch using intraoperative ultrasound or with 1 ml of ICG (2.5 mg/ml) systemically after blocking the Glissonean pedicle of the tumor. These processes are referred to as positive staining (**Video 2**) and negative staining (**Video 3**), respectively. After application of the exclusion criteria and 1:1 PSM, 81 ICG-guided LH and 81 CLH cases were compared. The study complies with the Declaration of Helsinki and was reviewed and approved in writing by the Ethics Review Institution of Guangdong Provincial People’s Hospital, and an application to the ethics committee for waiver of patient informed consent has been made.

### Inclusion and exclusion criteria

The patient inclusion criteria was as follows: (I) a diagnosis of HCC according to postoperative pathology and (II) operation performed laparoscopically. The exclusion criteria were as follows: (I) patients who had undergone surgical hepatectomy before confirmation of postoperative pathology for HCC, (II) conversion from LH to open hepatectomy and (III) non-radical resection. Patients who had (IV) incomplete information were also excluded.

### Data collection

The information that was obtained from patients in this study included baseline information, intraoperative conditions, pathology and postoperative conditions. The baseline data that was collected included sex, age, weight, preoperative treatment, comorbidities, evaluation of New York Heart Association (NAYA), American Society of Anesthesiologists (ASA), Child-Pugh and Barcelona Clinic Liver Cancer (BCLC), tumor size, tumor number, hepatitis, cirrhosis, ascites, portal hypertension, tumor thrombus, total bilirubin, albumin, alpha-fetoprotein and prothrombin time. Intraoperative conditions included the surgical method, blood loss, operative time and blood transfusion rate. Pathology included the degree of differentiation, surgical margin, cirrhosis, macrovascular invasion and microvascular invasion (MVI). Postoperative conditions included postoperative complications, length of stay, mortality, postoperative treatment, overall survival (OS) and RFS. Most information came from the physician management system, consisting of hospital records, surgical records, anesthesia records, doctors’ advice, imaging results, etc. Information on OS and RFS was obtained from regular follow-up by telephone.

### Data definition

Patients treated with hepatic arterial chemoembolization, local ablation therapies, targeted therapy or immunity therapy before surgery were recognized as having undergone preoperative treatment Patients with an additional malignant tumor were defined as those with a history of tumors. Tumor size was defined as a long trial when there was only one isolated tumor or an add-up long trial when there were two or more isolated tumors measured by videography before surgery. Cirrhosis, tumor thrombus, ascites and portal hypertension were also assessed using videography. The NAYA and ASA classifications were evaluated in anesthesia records by anesthetists. Blood loss and operative time were accurately recorded in anesthesia records. Surgical methods and blood transfusion rates were checked in surgical records. OS was defined as the time from surgery to death or last follow-up. RFS was defined as the time from surgery to the day recurrence was diagnosed using videography.

### Propensity score matching

Univariate analysis was used to assess the baseline comparability of the two groups. Multivariate analysis was used to identify possible factors contributing to OS and RFS. Unbalanced baseline and possible factors according to the results of univariate analysis and multivariate analysis were put into the 1:1 PSM. This was conducted using logistic regression according to a “optimal-neighbor matching” method. After 1:1 PSM, the baseline and mentioned possible factors were re-evaluated between the two groups.

### Statistical analyses

Statistical analyses were conducted using Statistical Product and Service Solutions (SPSS) version 26.0. OS and RFS charts were drawn using GraphPad Prism version 9.3.1.471. In descriptive statistics, categorical data are presented as absolute numbers and proportions, while continuous variables are presented as medians and quartiles. For hypothetical statistics, categorical data were compared using the Pearson chi-square test, continuity correction or Mann-Whitney U test, while continuous variables were compared using the Mann-Whitney U test. OS and RFS were analyzed using the Kaplan-Meier method and compared using the log-rank test. Statistical significance was set at P < 0.05.

## Results

### Baseline characteristics before PSM

From January 2014 to December 2020, 289 laparoscopic hepatectomies were performed for HCC management. After exclusion, 104 ICG-guided LH and 158 CLH cases were statistically compared. Before 1:1 PSM, baseline characteristics were comparable between the groups, apart from albumin, which was less in the CLH group compared with the ICG-guided LH group [37.75 (35.49-40.13) vs. 39.25 (36.73-41.75); p=0.008]. For Child-Pugh classification, more patients presented with Child-Pugh B or C in the CLH group than in the ICG-guided LH group [n=13 (8.20%) vs. n=0 (0%); p=0.003]. There were also more patients with hepatitis C in the CLH group than in the ICG-guided LH group [n=18 (11.4%) vs. n=4 (3.8%); p=0.031]. Regarding the degree of differentiation [p=0.016] and postoperative treatment, there were more patients with postoperative treatment in the CLH group than in the ICG-guided LH group [n=65 (41.1%) vs. n=22 (21.2%); p=0.001]. Finally, there were more patients with postoperative Transcatheter Arterial Chemoembolization (TACE) or Hepatic Artery Infusion Chemotherapy (HAIC) in the CLH group than in the ICG-guided LH group [n=49 (31%) vs. n=11 (10.6%); p=0.000]. The details are presented in **Table 1**.

**Table 1:** Comparison between baseline characteristics.

	unmatched cohort			1:1 Propensity score matching		
	**LH**	**ICG-guided**	**p value**	**LH**	**ICG-guided**	**p value**
	**(n=158)**	**LH (n=104)**		**(n=81)**	LH (n=81)	
Sex, n (%)			0.681			0.828
Male	138 (87.3)	89 (86.6)		69 (85.2)	68 (84)	
Female	20 (12.7)	15 (13.4)		12 (14.8)	13 (16)	
Age, n(%), years			0.092			0.397
≤49	52 (32.9)	28(26.9)		26 (32.1)	25 (30.9)	
50-59	49 (31)	25 (24)		25 (30.9)	17 (21)	
60-69	38 (24.1)	36 (34.6)		20 (24.7)	28 (34.6)	
≥70	19 (12)	15 (14)		10 (12.3)	11 (13.6)	
Weight, median (IQR), kg	65 (59.8- 70.3)	64 (56-70)	0.238	66 (60-71.8)	64 (57-70)	0.125
Preoperative treatment						
TACE or HAIC, n(%)	7 (4.4)	4 (4.9)	1	3 (3.7)	4 (4.9)
local ablation, n(%)	2 (1.3)	1 (0.96)	0.67	0 (0)	0 (0)	1
Comorbidities						
Hypertension,n(%)	37 (23.4)	27 (26)	0.639	17 (21)	22 (27.2)	0.358
Diabetes, n(%)	19 (12)	11 (10.6)	0.719	10 (12.3)	9 (11.1)	0.807
Coronary heart disease, n(%)	4 (2.5)	3 (2.9)	1	1 (1.2)	3 (3.7)	0.613
History of tumor, n(%)						
	6 (3.8)	3 (2.9)	0.96	4 (4.9)	2 (2.5)	0.677
Evaluation						
NYHA, n(%)			0.604			0.396
I	103 (65.2)	64 (61.5)		55 (67.9)	49( 60.5)	
II	53 (33.5)	40 (38.5)		24 (29.6)	32 (39.5)	
III	2 (1.3)	0 (0)		2 (2.5)	0 (0)	
ASA, n(%)			0.112			0.228
I	25 (15.8)	11 (10.6)		16 (19.8)	8 (9.9)	
II	124 (78.5)	83 (79.8)		58 (71.6)	67,(82.7)	
III	9 (5.7)	10 (9.6)		7 (8.6)	6(7.4)	
Child-Pugh, n(%)			0.003			1
A	145 (91.8)	104 (100)		81 (100)	81 (100)	
B	12 (7.6)	0 (0)		0 (0)	0 (0)	
C	1(0.6)	0 (0)		0 (0)	0 (0)	
BCLC, n(%)			0.318			0.467
0	27 (17.1)	15 (14.4)		15 (18.5)	11 (13.6)	
A	124 (78.5)	81 (77.9)		62 (76.5)	66 (81.5)	
B	6 (3.8)	7 (6.7)		3 (3.7)	3 (3.7)	
C	1 (0.6)	1 (1.0)		1 (1.2)	1 (1.2)	
Liver condition						
Hepatitis B, n(%)	129 (81.6)	93 (89.4)	0.087	74 (91.4)	73 (90.1)	0.786
Hepatitis C, n(%)	18(11.4)	4 (3.8)	0.031	4 (4.9)	4 (4.9)	1
Imaging Cirrhosis, n(%)	62 (39.2)	31 (29.8)	0.118	26 (32.1)	27 (33.3)	0.867
Portal hypertension, n(%)	26 (16.5)	17 (16.3)	0.981	9 (11.1)	15 (18.5)	0.185
Tumor thrombus, n(%)	1(0.6)	1 (1)	1	1 (1.2)	1 (1.2)	1
Ascites, n(%)	3 (1.9)	3(2.9)	0.92	0 (0)	3 (3.7)	0.244
Tumor condition						
Tumor size, median (IQR), mm	32.5 (23-47)	35.5 (23-53)	0.401	30 (22-50.5)	35 (23-54)	0.354
<50	122 (77.2)	73 (70.2)	0.241	59 (72.8)	57 (70.4)	0.811
50-100	32 (20.3)	30 (28.8)		19 (23.5)	23 (28.4)	
>100	4 (2.5)	1 (1)		3 (3.7)	1 (1.2)	
Tumor number, n(%)						0.213
1	151 (95.6)	93 (89.4)	0.059	78 (96.3)	74 (91.4)	
2	4 (2.5)	9 (8.7)		1 (1.2)	2 (8.6)	
3	1 (0.6)	0 (0)		0 (0)	0 (0)	
>3	2 (1.3)	2 (1.9)		2 (2.5)	0 (0)	
Grade, n(%)			0.016			0.88
Well-differentiated	9 (5.7)	6 (5.8)		7 (8..6)	4(4.9)	
Moderately differentiated	147 (93)	98 (94.2)		44 (54.3)	48 (59.3)	
Poorly differentiated	2 (1.3)	0 (0)		30 (37)	29 (35.8)	
Macrovascular invasion, n(%)	0 (0)	1 (1)	0.833	0 (0)	1 (1.2)	1
Microvascular invasion, n(%)	30 (19)	17 (16.3)	0.586	14 (17.3)	16 (19.8)	0.686
Pathologically Cirrhosis , n(%)	82 (51.9)	46 (44.2)	0.224	43 (53.1)	40 (49.4)	0.637
Postoperative treatment						
Treatment, n(%)	65 (41.1)	22 (21.2)	0.001	20 (24.7)	2 0(24.7)	1
Surgery, n(%)	17 (10.8)	6 (5.8)	0.163	4 (4.9)	6 (7.4)	0.514
TACE or HAIC, n(%)	49 (31)	11 (10.6)	0	13 (16)	11 (13.6)	0.658
Drugs, n(%)	6 (3.8)	2 (1.9)	0.62	0 (0)	0(0)	1
Total bilirubin, median (IQR),umol/L	15.0(11.5-18.8)	14.1 (10.7-17.7)	0.12	15.1 (11.7-18.7)	13.4 (10.5-18.0)	0.136
Albumin, median (IQR), g/L	37.8 (35.5-40.1)	39.3 (36.7-41.8)	0.008	38.9 (36.8-40.8)	39 (35.5-41.3)	0.758
Alpha-fetoprotein, median (IQR), ng/ml	24.8 (4.2-275.1)	15.0 (4.2-280.9	0.632	14.2 (3.8-311.7)	16.1 (4.3-273.7)	0.769
Prothrombin time, median (IQR), sec	13.8 (13.2-14.4)	13.8 (13.2-14.4)	0.868	13.8 (13.2-14.5)	13.8 (13.2-14.7)	0.619
Surgical method, n(%)			0.424			0.452
Right hepatectomy	6 (3.8)	2 (1.9)		4 (4.9)	2 (2.5)	
Left hepatectomy	5 (3.2)	4 (3.4)		3 (3.7)	3 (3.7)	
Extended left hepatectomy	1 (0.6)	1 (1.0)		1 (1.2)	0(0)	
Left lateral sectionectomy	13 (8.2)	9 (8.7)		9 (11.1)	4 (4.9)	
Anatomical segmentectomy	1 (0.6)	9 (8.7)		1 (1.2)	7 (8.6)	
Right anterior hepatectomy	1 (0.6)	1 (1.0)		0 (0)	0 (0)	
right posterior hepatectomy	0 (0)	2 (1.9)		0 (0)	1 (1.2)	
Wedge resection	131 (82.9)	76 (73.1)		63 (77.8)	64 (50.4)	

^∗^Statistical significance was set at P < 0.05.

### Univariate and multivariate analysis of OS and RFS

Before 1:1 PSM, univariate analysis showed that the degree of differentiation (P=0.003) and targeted therapy or immunotherapy (P=0.047) were correlated with OS. Sex (P=0.009), age (P=0.031), alpha-fetoprotein (P=0.005), HBV (P=0.046), postoperative treatment (P=0.003), postoperative surgery (P=0.041), tumor size (P=0.046) and MVI (P=0.005) were correlated with RFS.

### Baseline characteristics after PSM

Sex, age, degree of differentiation, AFP, ALB, HBV, HCV, Child-Pugh score, tumor number, surgical method, MVI, postoperative treatment, postoperative surgery, postoperative TACE or HAIC and postoperative drugs were included as predictors in the PSM. After 1:1 PSM, 81 ICG-guided LH and 81 CLH were compared. Baseline characteristics were comparable between the 81 ICG-guided LH and 81 CLH groups. The details are showed in **Table 1**.

### Comparison of short-term outcomes and survival results after PSM

The baseline characteristics of both groups were comparable after matching. The statistical details of short-term outcomes are shown in **Table 2**. There was a statistically significant difference in operative time that was longer in the ICG-guided LH group than in the CLH group [268 min (222.5-322.5) vs 230 min (167.5-285); p=0.004]. There was no significant difference in operative time in anatomical resection between the two groups (p=0.987), while there was a significant difference in operative time in non-anatomical resection that was shown to be longer in the ICG-guided LH group than in the CLH group (p=0.001). There were no significant difference of positive surgery margin [n=1 (1.2%)] in the CLH group compared with that in the ICG-guided LH group (p=1), nor were there significant differences in blood loss [100 ml (50-450) in the CLH group versus 200 ml (100-400) in the ICG-guided LH group (p=0.319)], blood transfusion rate [n=9 (11.1%) in the CLH group versus n=5 (6.2%) in the ICG-guided LH group (p = 0.263)], postoperative bleeding [n=0 (0%) in the CLH group versus n=2 (2.5%) in the ICG-guided LH group (p=0.477)], bile leakage [n=0 (0%) in the CLH group versus n=0 (0%) in the ICG-guided LH group (p=1)], liver failure [n=2 (2.5%) in the CLH group versus n=2 (2.5%) in the ICG-guided LH group] and postoperative length of stay [7 days (6-8) in the CLH group versus 7 days (6-10) in the ICG-guided LH group (p=0.081)]. There was also no significant difference in mortality within 30 days between the two groups [n=0 (0%) in the CLH group versus n=1 (1.2%) in the ICG-guided LH group (p=1) and mortality within 90 days [n=1 (1.2%) in the CLH group versus n=2 (2.5%) in the ICG-guided LH group (p =1)].

**Table 2:** Comparison between short-term outcomes and survival outcomes.

	unmatched cohort			1:1 Propensity score matching		
	**LH**	**ICG-guided**	**P value**	**LH**	**ICG-guided**	**p value**
	**(n=158)**	**LH (n=104)**		**(n=81)**	**LH (n=81)**	
Surgery margin, n (%)	1 (0.6)	2 (1.9)	0.714	1 (1.2)	1 (1.2)	1
Operative time, median (IQR),min	229.5 (180-285.8)	268 (211.3-328.8)	0.008	230 (167.5-285)	268 (222.5-322.5)	0.004
	
anatomical resection						0.9870
non-anatomical resection						0.001
Blood loss						
Blood loss, median (IQR),ml	100 (50-400)	175 (50-400)	0.159	100 (50-450)	200 (100-400)	0.319
						
<400ml	123 (77.8)	80 (76.9)	0.861	61 (75.3)	63 (77.8)	
>400ml	35 (22.2)	24 (23.1)		20 (24.7)	18 (22.2)	0.711
Blood transfusion rate, n (%)	18 (11.4)	8 (7.7)	0.327	9 (11.1)	5 (6.2)	0.263
Postoperative length of stay, median (IQR),days	7 (6-9)	7 (6-10)	0.675	7 (6-8)	7 (6-10)	0.081
Complication						
bleeding, n (%)	2 (1.3)	3 (2.9)	0.634	0 (0)	2 (2.5)	0.477
Biliary fistula, n (%)	1 (0.6)	1 (1)	1	0 (0)	0 (0)	1
Liver failure, n (%)			0.143			0.333
PHLF A	4 (2.5)	0 (0)		2 (2.5)	0 (0)	
PHLF B	1 (0.6)	0 (0)		0 (0)	0 (0)	
PHLF C	1 (0.6)	2 (1.9)		0 (0)	2 (2.5)	
Clavien-Dindo			0.643			0.141
Classification 1	9 (5.7)	5 (4.8)		2 (2.5)	3 (3.7)	
Classification 2	0 (0)	1 (1.0)		0 (0)	1 (1.2)	
Classification 3	0 (0)	1 (1.0)		0 (0)	1 (1.2)	
Classification 4	1 (0.6)	1 (1.0)		0 (0)	1 (1.2)	
Prognosis						
Mortality within 30 days, n (%)	2 (1.3)	1 (1)	1	0 (0)	1 (1.2)	1
Mortality within 90 days, n (%)	3 (1.9)	2 (1.9)	1	1 (1.2)	2 (2.5)	1
1-year RFS, (%)	0.797	0.874		0.816	0.865	
2-year RFS, (%)	0.696	0.711		0.756	0.697	
3-year RFS, (%)	0.624	0.627		0.72	0.587	
4-year RFS, (%)	0.569	0.522		0.679	0.44	
1-year OS, (%)	0.922	0.972		0.935	0.961	
2-Year OS, (%)	0.865	0.937		0.908	0.922	
3-Year OS, (%)	0.789	0.915s		0.809	0.896	
4-Year OS, (%)	0.769	0.832		0.77	0.806

^∗^Statistical significance was set at P < 0.05.

### Overall and recurrence-free survival before and after PSM

**Figure 1:**
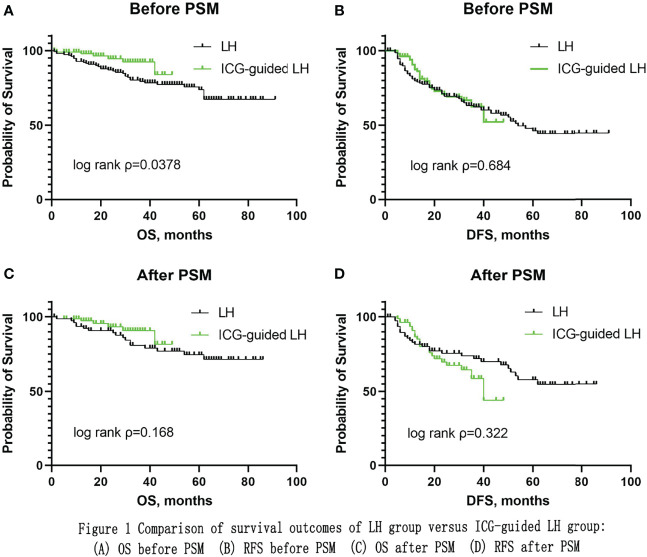
Comparison of survival outcomes of LH group versus ICG-guided LH group: **(A)** OS before PSM **(B)** RFS before PSM **(C)** OS after PSM **(D)** RFS after PSM.

Before 1:1 PSM, there was significant difference in OS (P=0.0378) while there was no significant difference in RFS (P=0.684) between the two groups (**Figure 1**). After 1:1 PSM, the median follow-up time was 46 months in the CLH group and 26 months in the ICG-guided LH group (P=0.000). The ICG-guided LH group appeared to have a trend towards better OS, but there was no significant difference in OS (P=0.168) and RFS (P=0.322) between the two groups (**Figure 1**). The 1-, 2-, 3- and 4-year OS rates were 93.5%, 90.8%, 80.9% and 77%, respectively, in the CLH group and 96.1%, 92.2%, 89.6% and 80.6%, respectively, in the ICG-guided LH group. The 1-, 2-, 3- and 4-year RFS rates were 81.6%, 75.6%, 72.0% and 67.9%, respectively, in the CLH group and 86.5%, 69.7%, 58.7% and 44%, respectively, in the ICG-guided LH group.

## Discussion

Laparoscopic liver resection has been shown to be a safe and minimally invasive surgical procedure (19). Fluorescence laparoscopy has overcome difficulties associated with laparoscopic surgery, such as tumor localization for the treatment of liver malignant tumors and is expected to improve perioperative indicators and long-term prognosis (20, 21).

### Indocyanine green fluorescence aids intraoperative tumor identification

Hepatocytes can take up and excrete ICG, and the tumor site is stained owing to abnormal excretion; therefore, the tumor can be specifically visualized (22). By intravenous injection of 0.5 mg/kg ICG 3-7 days before surgery, a circle of fluorescence around the liver cancer can be seen during surgery, which aids in immediately identifying superficial tumors (23, 24). Compared with intraoperative ultrasound, ICG fluorescence can help the chief surgeon find the specific location of the tumor faster and determine the extent of tumor resection (25).

Previous studies by our team have shown that fluoroscopy provides more precise information on tumor location than intraoperative ultrasound, particularly for sites of difficult liver segments (24). Although there are no valid statistical data in this study to prove the localization effect of ICG, such as shortening the operation time, in some cases we observed some lesions that were not detected on preoperative imaging. After surgical resection of these lesions, the postoperative pathology report was HCC. We are greatly encouraged by the ability of fluoroscopy to detect imaging-negative HCC, demonstrating that fluoroscopy is a promising laparoscopic technique. In cases of small superficial tumors, fluoroscopy can quickly identify the lesions during the operation, which greatly shortens the operation time.

### Indocyanine green fluorescence helps reduce intraoperative bleeding

Blood loss during laparoscopy is greatly reduced compared with that during open surgery (26, 27). Through preoperative three-dimensional reconstruction and intraoperative ICG, the relationship between large blood vessels and tumors can be fully identified and the possibility of major bleeding can be reduced, thereby reducing intraoperative bleeding (28). Intraoperative blocking of the hepatic hilum can reduce intraoperative bleeding and clarify the operative field (29, 30). Other laparoscopic techniques, including appropriate pneumoperitoneum pressure and low central venous pressure, can also reduce intraoperative bleeding (31, 32).

Previous studies have reported that ICG can reduce intraoperative blood loss (15). Herein, we report a case that supports these results. Preoperative enhanced CT showed malignant lesions in the S4 and S2 segments of the liver, with diameters of 6 cm and 5 cm, respectively. The surgery was performed on February 27, 2018. Intraoperative exploration was consistent with preoperative imaging, and left hepatectomy was performed. The left hepatic pedicle was separated and clipped during the operation and the ICG was injected peripherally. The S5, S6, S7, and S8 segments were stained green. Left hepatectomy was performed according to the liver staining band, and intraoperative blood loss was only 80 ml. The patient survived until the last follow-up on April 30, 2021.

Our study shows that fluorescein laparoscopy is a safe laparoscopic technique with low intraoperative bleeding and intraoperative blood transfusion rates.

### ICG-guided resection causes fewer severe liver failures

Postoperative primary liver failure is the leading cause of death following hepatectomy (33). Risk factors include underlying liver disease, the extent of resection and intraoperative conditions (33). Before major hepatectomy, functional and volumetric assessments of the remnant liver are critical (34). Lack of preoperative residual liver assessment and excessive intraoperative bleeding can increase the incidence of postoperative primary liver failure (35). Large hepatectomy requiring removal of the portal vein often leads to postoperative liver failure and increases perioperative mortality, especially in patients with hepatic steatosis (36).

Fortunately, compared with traditional LH, fluoroscopy-guided LH for non-anatomical hepatectomy can ensure both R0 resection (15) and a safe margin within the 6-8 mm range (7, 37), avoiding the larger resection range brought about by anatomical hepatectomy.

In the present study, 82.9% of 262 patients underwent non-anatomical hepatectomy with conventional laparoscopy, 73.1% underwent non-anatomical hepatectomy with fluorescence laparoscopy and 98.8% underwent negative surgery margin. Only three patients (1.1%) developed severe postoperative liver failure, which was reported at low levels (1%-9%) in the literature (38); two of the three underwent anatomical hepatectomy.

### ICG-guided non-anatomical resection may ensure safe surgery margin and lead to improved OS

Previous studies on HCC and fluorescence laparoscopy have rarely addressed prognosis. We know that the fluorescence border seen during surgery is not equal to the tumor border but is wider than the tumor border. This provides R0 resection and a wide incisal margin. In recent years, wide resection margins for hepatic malignancies have been associated with a better prognosis (39–42). Fluoroscopic resection of HCC is expected to improve prognosis. In the present study, the fluorescence laparoscopy group appeared to have better OS.

### Limitation

Our study had some limitations. Firstly, the follow-up time of the ICG-guided LH group was not long enough, resulting in a significant difference in the follow-up time between the two groups. Secondly, we collected data retrospectively, and the retrospective study was more biased. Thirdly, the sample size was small; increasing the sample size may make the results statistically different. Fourthly, the number of positive events was small. For example, the number of cases of bile leakage was small, which resulted in the differences being insignificant.

## Conclusion

Although ICG fluorescence-guided LH is a timelier procedure to perform, it is a safe and effective technique with the advantages of intraoperative positioning, low postoperative complication rates and the potential to improve OS.

## Data availability statement

The raw data supporting the conclusions of this article will be made available by the authors, without undue reservation.

## Ethics statement

Ethical approval was not provided for this study on human participants because Retrospective analysis may apply for exemption from ethical review. Written informed consent for participation was not required for this study in accordance with the national legislation and the institutional requirements.

## Author contributions

WJ and ZX collected the related data and completed the manuscript and figures. ZZH, ZZ, and PT did the statistical analysis. LY, JH, JZ, and WH did the operations. WH gave constructive guidance and made critical revisions of the manuscript. WJ and WH participated in the design of this paper. All authors contributed to the article and approved the submitted version.

## Conflict of interest

The authors declare that the research was conducted in the absence of any commercial or financial relationships that could be construed as a potential conflict of interest.

## Publisher’s note

All claims expressed in this article are solely those of the authors and do not necessarily represent those of their affiliated organizations, or those of the publisher, the editors and the reviewers. Any product that may be evaluated in this article, or claim that may be made by its manufacturer, is not guaranteed or endorsed by the publisher.
